# A Conundrum

**Published:** 1907-07

**Authors:** 


### EDITORIAL NOTES AND COMMENTS.

The Business Office of the JOURNAL-RECORD is 1 1-2 to 5 1-2 South Broad Street.

The Editorial Office is 318-319-320 The Prudential.

Address all Business Commucications to Dr. George Brown, Manager.

Make remittances payable to THE JOURNAL-RECORD OF MEDICINE.

On matters pertaining to the Editorial and Original communications, address Dr, Bernard Wolff, Atlanta.

Reprints of original articles will be furnished at cost price. Requests for the same should always be made on THE MANUSCRIPT.

We will present, postpaid, on request, to each contributor of an original article, twenty (20) marked copies of THE JOURNAL-RECORD OF MEDICINE containing such article.

### A CONUNDRUM.

Founded on human nature and ethics—courtesy.

A gentleman's remarks to me to-day in my consultation room made me quickly turn to him and rather decidedly say: "What! I am surprised at such a question from you!"

Now what was it that this bright young man and up-to-date doctor said to me? This is the question:—

He said, "Dr., I was frightened, I was miserable, but now my eye is well and I am happy, how much do I owe you?"

The above conundrum was sent us by Arthur G. Hobbs to carry a point on the ethics and courtesy, that so many young men in our profession do not fully understand or comprehend.

